# Clinical competency of nurses trained in competency-based versus objective-based education in the Democratic Republic of the Congo: a qualitative study

**DOI:** 10.1186/s12960-024-00921-0

**Published:** 2024-06-04

**Authors:** Mari Nagai, Miyuki Oikawa, Tomoko Komagata, Josué Désiré Bapitani Basuana, Gérard Kahombo Ulyabo, Yui Minagawa, Sadatoshi Matsuoka, Yuriko Egami, Mari Honda, Toyomitsu Tamura

**Affiliations:** 1 Bureau of International Health Cooperation, National Center for Global Health and Medicine, Tokyo, Japan; 2 National College of Nursing, National Center for Global Health and Medicine, Tokyo, Japan; 3 School of Nursing, Tokyo Women’s Medical University, Tokyo, Japan; 4 Department of Health Science Education, Ministry of Public Health, Kinshasa, Democratic Republic of the Congo

**Keywords:** Clinical nurses, Clinical supervisors, Competency-based education, Democratic Republic of the Congo, Resource-limited countries, Standardized nursing care

## Abstract

**Background:**

Designing competency-based education (CBE) programmes is a priority in global nursing education for better nursing care for the population. In the Democratic Republic of the Congo (DRC), object-based education (OBE) remains mainstream in pre-service nursing education programmes. Recently, the Ministry of Health developed a self-assessment tool and quantitatively compared the clinical competency of CBE- and OBE-trained nurses. This study aimed to qualitatively triangulate the results of self-evaluation by exploring perception of supervisors, incumbent CBE-, and OBE-trained nurses in comparison with the competence of the two types of nurses, and to identify influential factors or barriers to their competence in clinical settings.

**Methods:**

A qualitative descriptive approach with conventional content analysis was applied. Twenty interviews with clinical supervisors who oversaw both CBE- and OBE-trained nurses, 22 focus group discussions (FGDs) with CBE-trained nurses, and 21 FGDs with OBA-trained nurses currently working in health facilities were conducted. Participants of the FGDs were selected from the participants of the DRC self-assessment competency comparison study where there was no statistically significance between CBE- and OBE-trained nurses in the demographic characteristics. Data were analysed in terms of the competencies identified by the Ministry of Health.

**Results:**

The supervisors recognised that the CBE-trained nurses had stronger competencies in professional communication, making decisions about health problems, and engaging in professional development, but were weak in clinical skills. This study identified challenges for supervisors in assuring standardised care in health facilities with OBE- and CBE-trained nurses, as well as barriers for CBE-trained nurses as a minority in the workplace in demonstrating their competencies.

**Conclusions:**

The study results support the Ministry of Health’s policy to expand CBE in pre-service education programmes but reveal that its slow implementation impedes full utilisation of the acquired competencies at health facilities. Implementation could be accelerated by strengthening cooperation among the Ministry of Health’s three human resource departments, and developing and implementing a well-planned, legally binding, long-term CBE reform strategy, including an approach to the Continuing Professional Development system.

**Supplementary Information:**

The online version contains supplementary material available at 10.1186/s12960-024-00921-0.

## Background

Primary healthcare (PHC) is the cornerstone of universal health coverage (UHC). Its implementation requires a diverse workforce, including nurses who have acquired competencies to address people’s health needs [1]. As an outcome-based and learner-centric approach, competency-based education (CBE) enhances the clinical performance of healthcare providers [2]. CBE is expected to produce a health workforce that can provide optimal care by making comprehensive decisions based on acquired competencies [3]. The shift from traditional object-based education (OBE) to CBE has been a key trend in health professional education worldwide [4, 5]. The Global Strategic Direction for Nursing and Midwifery also identifies CBE as a priority in creating policies in the global nursing education sector [6].

The achievement of UHC is a salient health policy in the Democratic Republic of the Congo (DRC) [7]. There, PHC is primarily available in health districts where health centres (HCs) provide frontline health services and general hospitals (GHs) serve as referrals. Most of the health service provisions in health facilities, especially at HCs, rely on nurses, as the health workforce regulation does not require allocation of a doctor due to shortages. Pre-service nursing education is provided through different systems by two ministries; the Ministry of Higher and University Education governs bachelor’s and advanced diploma courses in nursing education, while the Ministry of Public Health (MoH) governs secondary nursing education institutions and certificate courses for secondary nurses. As of 2019, nurses accounted for 47.1% (93,218) of all health personnel registered by the MoH, of which 37.0% (34,449) of nurses were secondary nurses [8]. While primary nurses are expected to work in health administration or take managerial responsibility in health facilities, secondary nurses play a critical role in the DRC to perform multiple nursing tasks, including providing PHC in lower-level health facilities [9]. Based on a 2002 survey which identified the gap between the outcomes of pre-service secondary nursing education and the competencies required for clinical practice [7], the MoH issued a ministerial decree in 2005 for introducing CBE in secondary nursing education institutions to strengthen pre-service education and provide secondary nurses with the competencies required for providing PHC [10]. Twenty-six decentralised provincial health departments are responsible for managing the health workforce, including secondary nursing education institutions. As of 2019, only 110 (21%) of the 526 secondary nursing education institutions nationwide implement CBE [8]. Despite support from external partners such as a nursing education partnership initiative to expand access to CBE programmes [11], seven out of the 26 provinces have not introduced CBE at all. In the provinces that partially introduced CBE, some nursing education institutions continued to offer OBE simultaneously. Thus, in some HCs and GHs, both newly qualified CBE- and OBE-secondary nurses work together. CBE implementation challenges have been reported in the DRC and other African countries, with obstacles including lack of teachers, capital, and planning [11, 12].

Secondary nurses in the DRC must have five competencies for clinical practice: (1) establishing professional communication, (2) making decisions about health problems, (3) performing nursing interventions, (4) managing resources, and (5) engaging in professional development. Nursing students must acquire skills such as collecting patient data, identifying the patient's health problems, and planning a nursing care plan [13]. The scope of CBE goes beyond mere skill development; it entails mastering comprehensive nursing skills [5]. It integrates acquired knowledge, skills, and attitudes, which can enhance clinical performance [14–21]. The CBE programmes in secondary nursing education institutions allocate 358 h to integrate vertical knowledge and skills into optimal care over four years, whereas OBE programmes do not provide this opportunity [22].

After more than 10 years since CBE-trained secondary nurses started working in health facilities in the DRC, the MoH developed a DRC self-assessment competency scale and compared the competencies of CBE-trained with those of OBE-trained secondary nurses, both having 2–5 years of clinical experience. The result showed that the former has statistically higher competency in communication, decision making, and nursing interventions [23]. However, it was unclear how their supervisors perceive their competence, and how these two types of nurses interact with each other in clinical settings [24]. This study aimed to qualitatively triangulate the results of self-evaluation by exploring perception of supervisors, incumbent CBE-, and OBE nurses in comparison with the competence of the two types of nurses, as well as to identify influential factors or barriers to their competence in clinical settings.

## Methods

### Design, setting, and participants

A qualitative descriptive approach with conventional content analysis was applied to achieve the aims of the study. The target population and the selection criteria were: (1) secondary nurses with 2–5 years of clinical experience, who had graduated after 2012 from nursing education institutions using CBE in the DRC and currently worked in lower level health facilities such as HCs or GHs providing PHC (CBE-nurses); (2) secondary nurses with 2–5 years of clinical experience, who had graduated after 2012 from nursing education institutions using OBE and currently worked in lower level health facilities such as HCs or GHs providing PHC (OBE-nurses); and (3) clinical supervisors who oversaw both the CBE- and OBE-nurses in the health facilities. For this qualitative study, we randomly selected CBE- and OBE-nurses from participants of the DRC self-assessment competency comparison study [23] where there was no statistically significance between the two groups in demographic characteristics such as gender, age, years of experience, type of health facility, and position in the health facilities. For the comparison study, nine of the 26 provinces in DRC (Sud-Kivu, Nord-Kivu, Kasai-Central, Kongo-Central, Lualaba, Haut-Katanga, Kwilu, Kasai-Oriental, and Kinshasa) were selected based on transport accessibility and safety considerations for the research team to visit from Kinshasa [25]. Next, to compare OBE and CBE in similar conditions, a total of 10 cities (two from Kwilu and one from other 8 provinces) were selected where nursing education institutions applying OBE and CBE co-exist in both urban and rural areas, using the graduates list provided by the MoH. Then, health facilities where those two types of graduates were working were identified with the help of each city’s education institutions and provincial and district health offices. We identified the clinical supervisors who oversaw both CBE- and OBE- nurses in those health facilities.

Study participants had to be accessible at the time of the study, be able to travel to the interview site, and provide their consent to participate in the study. Most clinical supervisors were trained in OBE when they were nursing students. Sampling was discontinued when theoretical saturation was reached.

### Data collection

Open-ended questionnaires were administered. Individual interviews were conducted for supervisors, while focus group discussions (FGDs) were conducted with CBE- and OBE-nurses separately. The research team (six authors) who had strong local contextual knowledge and experience in research in low- and middle-income countries including the DRC developed interview guides for the individual interviews and FGDs in French. The interview guides comprised questions regarding respondents’ demographics, their perceptions of the competence of secondary nurses in general, the differences between the competence of CBE- and OBE-nurses, and their views on improving nursing care. The questionnaires and guides were pilot-tested and finalised. Prior to data collection, the chief officer of the Department of Health Science Education in the MoH trained 10 MoH officers and two provincial health officers per province on the interview guide to conduct quality interviews in the provinces. Between January and September 2021, 10 interview teams (eight of which were gender-mixed), each comprising one MoH officer from Kinshasa and two officers from the target provinces, collected data. All interviews and FGDs were conducted face-to-face in a private setting at nursing education institutions or district health offices to ensure a favourable and private interview environment. Each interview and FGD lasted between 30–60 min and were recorded using an audio recorder. Participants were informed of their right to refuse study participation and assured of the confidentiality of the information they provided. They were paid travel allowances from their workplace to the interview venue. Twenty supervisors were interviewed (11 males, 9 females, 10 working at public health facilities, 2 in private health facilities, 5 in religious health facilities and 3 in others), and 22 FGDs with CBE-nurses and 21 FGDs with OBE-nurses (6–8 participants per each FGD) were conducted.

### Ethical consideration

The authors obtained ethical approval from the Ethics Committees of the MoH of the DRC (No. 137/CNES/BN/PMMF/2019 du 21/09/2019) and the National Center for Global Health and Medicine in Japan (NCGM-G-004023-00). All participants provided written informed consent.

### Data analysis

The authors transcribed the audio-recorded interviews into Microsoft Word in French, then manually analysed the transcripts and read and reread them to familiarise themselves with the data. In accordance with the qualitative descriptive methodology, perceptions of the differences between CBE and OBE nurses and challenges in health facilities were identified, extracted and coded. A total of 26 codes were further analysed to identify similarities and differences, then categorised into nine themes and matched with the five competencies required for clinical practice, as well as influential factors or barriers to competency in the clinical setting.

### Trustworthiness

Credibility of the findings was enhanced through data source, methodological and investigator triangulations [26]. Data were collected from three sources, namely CBE-nurses, OBE-nurses, and their supervisors. Both individual interviews and FGDs were used to collect data. An individual interview allows the interviewee to speak freely and frankly and allows the interviewer to probe topics in certain depth without interruption [27]. FGDs are useful for assessment purposes because researchers can obtain wider opinions and perceptions from participants who build on each other’s ideas through ‘piggybacking’ [28]. During the interviews and FGDs, interviewers noted key points and restated them to participants to confirm accuracy or credibility. Furthermore, credibility was supported through the analysis and interpretation by all authors. The primary coding, categorisation of key phrases, and interpretation of the qualitative data were initially undertaken by the second author who had lived in the DRC for a significant period of time and had strong local contextual knowledge. The analysis was separately conducted by the first author with a qualitative research background of more than a decade, and the last author with the same background as the second author. These three authors presented initial analyses to the remaining authors to discuss interpretations and seek clarification and alternate explanations, which led to the enhancement of confirmability [26]. Transferability and dependability were boosted through description of the research context, and study procedures including data collection and analysis [26].

## Results

### Strength of CBE-nurses

Most of the supervisors recognised the CBE-nurses to be better, particularly at three competencies required for nurses in the DRC (i.e., establishing professional communication, making decisions about health problems, and engaging in professional development) which aligns with the findings of the quantitative study using the self-evaluation scale.

CBE allows nurses, in addition to the theory learned in school, to be confronted with realities on the ground... They try to solve problems in relation to the needs of the patients. (Supervisor 9 in Kasaï-Oriental Province)

Supervisors recognised that CBE-nurses communicate with patients and the community better than OBE-nurses, especially respecting patients and community habits, using understandable language, informing patients and communities about health services, and checking that given information was understood.

CBE-nurses communicate with patients before providing nursing care. They introduce themselves to the patient and start a conversation like ‘Where are you from?’ But OBE-nurses struggle to communicate with patients. (Supervisor 8 in Sud-Kivu Province)

OBE-nurses prescribe medicine and say, ‘Take it at home’. That’s it. CBE-nurses explain how to take the medicine, what the results will be, and observe whether the patient understood that information, then say ‘Come back to the health centre if you don’t feel better, I am happy to see how you are doing’. (Supervisor 8 in Kasaï-Central Province, Supervisor 8 in Sud-Kivu Province)

CBE-nurses go into the community, see how the community is suffering, and encourage them to visit the health centre. (Supervisor 8 in Kasaï-Central Province)

Supervisors also identified CBE-nurses’ superiority in data gathering from different sources to identify health problems, analysing them to plan nursing interventions, and assessing the results to improve the nursing plan. These are components to making decisions about health problems.

OBE-nurses are not interested in the root cause. CBE-nurses go out to understand the community and use that knowledge when they see patients at a health facility. (Supervisor 3 in Kwilu Province)

I found that CBE-nurses are very focused, can identify issues and priority needs of patients, and plan nursing care more effectively than OBE-nurses. (Supervisor 1 in Sud-Kivu Province)

However, some supervisors who did not know about the introduction of a competency-based programme criticised CBE-nurses as they try to share and discuss patients’ health issues with colleagues and supervisors, which is in fact one of the components of the competency to make decisions about health problems.

I find that the OBE-nurses can decide and work alone, but CBE-nurses always consult and involve other staff to make decisions. (Supervisor 7 in Sud-Kivu Province)

One supervisor identified the strength of CBE-nurses with their habit of active learning to update their knowledge, which is the competency to engage in professional development.

I think the education reform has done something. The CBE-nurses adapt better to the context and perform better in their duties. They are motivated and active. The CBE- nurses ask me questions to develop themselves, while OBE-nurses who graduated in the same year pretend they know everything, and don’t try to learn. (Supervisor 13 in Kongo Central Province)

### Weakness of CBE-nurses

Supervisors identified that CBE-nurses’ relative weakness is their clinical skills.

I don't know if it's because of the teacher or school curriculum, but I see that there is a problem with the CBE-nurses in terms of practical clinical skills. (Supervisor 2 in Lualaba Province)

I find that the CBE-nurses have some insufficiency, for example, they don't have the capacity of reading flowcharts or using partogrammes. I suspect that the school teacher or the supervisor of clinical practice did not teach enough about how to use those tools. (Supervisor 7 in Sud-Kivu Province)

FGDs separately organised with OBE- and CBE-nurses supported this supervisor’s observation about the novice of CBE nurses’ clinical skills.

CBE-nurses have shortcomings in practice. It seems they have not received enough practical training before graduation. For example, they are not good at attending delivery or calculating the doses of medicines and infusions for children. (FGD with OBE-nurses in Kinshasa)

Sometimes there are techniques that we still need to learn in-depth. Our knowledge and experience are limited. There are practices we didn’t confront during clinical training when we were students. (FGD with CBE-nurses in Bukavu province)

### Challenges in health facilities

Some supervisors recognise the difficulty of having OBE-nurses and CBE-nurses work together in the same health facility and the importance of the supervisor's role.

[With my mediation,] CBE- and OBE-nurses in my health facility share information to understand their differences and complement each other. (Supervisor 3 in Kwilu Province)

I ask CBE-nurses to mentor OBE-nurses so that the OBE-nurses can work like the CBE nurses. (Supervisor 8 in Kasaï-Central Province)

We, the supervisors, need to understand the new education, to be able to put ourselves in the shoes of those who give the current care [CBE-nurses] so that, between the two [OBE- and CBE-nurses], things can smoothly move forward. (Supervisor 3 in Haut-Katanga Province)

FGDs with the CBE-nurses supported the supervisor's statement that CBE-nurses were committed to improving care in healthcare facilities.

When I first came to work, OBE-nurses thought that I was going to fight with them. It was like a war. But by integrating them into everything I already knew, they are now able to provide care with the competency-based approach, too. I showed them that I am trained in five competencies in my school, and how to solve a problem by integrating several resources. (FGD with CBE-nurses in Bukavu province)

However, not all CBE-nurses have such positive experiences in their workplace. Rather, they struggle to get support and understanding from colleagues.

There are not a lot of CBE-nurses in my health facility. So, it's difficult... There are some people who accept my way of working, but others don't. They don’t know the new competency-based approach, so they criticise me. (FGD with CBE nurses in Kinshasa)

OBE-nurses criticised us saying that we are too proud because we studied with a competency base. They say we're going to replace them. We need to say, no, we didn’t come to replace you. (FGD with CBE nurses in Bukavu province)

Our way of working with the new approaches creates a conflict in the workplace. (FGD with CBE nurses in Kinshasa)

In such a work environment, CBE-nurses experience difficulties in utilising their competencies.

OBE-nurses tell me to follow their way. I’m the only CBE-nurse in my workplace. It's a difficult situation. ...Their work experience is greater than mine, so they say they know better than me. (FGD with CBE nurses in Kinshasa)

Where I work, the majority of the staff received OBE. They don’t know what I learned. Sometimes they treat me like a servant, and that causes conflicts between us. (FGD with CBE nurses in Kinshasa)

Based on such problematic situations in health facilities, both supervisors and CBE-nurses expressed the need for competency-based in-service training as recurrent programmes for experienced OBE-nurses/supervisors.

Most supervisors received OBE, so, they don’t know well about the competency-based approach. Training should be conducted for such supervisors so that all of us can have the same commitment. (Supervisor 1 in Sud-Kivu Province)

There is something we don't know about new education. When the education reform is applied at the school, we, who are in the health facilities, must also be briefed on this, to speak the same language with newly graduated nurses. (Supervisor 6 in Kasaï- Central Province)

I hope OBE-nurses get an opportunity to receive competency-based training. Then OBE- and CBE-nurses can work better together. (FGD with CBE nurses in Kinshasa)

Some supervisors noted that for CBE-nurses to demonstrate their competency, comprehensive health system strengthening is needed, including tackling the workforce shortage in health facilities.

Because of the shortage of staff, one nurse needs to cover several positions in my health facility and continuously work without the rest. We try to prioritise providing more or less satisfactory care for the patients. In this situation, when we find any issue in a nurse, it is difficult to judge if the nurse doesn’t have the competency, or is overwhelmed by the heavy workload. When the working conditions don't meet the standards, the judgement can be biased. Once the working condition meets the standards, then we can really reap the benefits of this new education approach in health facilities. (Supervisor 9 in Kasaï-Oriental Province)

## Discussion

Increasing the availability and quality of the health workforce and strengthening PHC are essential for achieving UHC in the DRC [7], and nurses are expected to play a particularly important role [9]. This is the first qualitative study to explore the effectiveness of CBE in secondary nurses who are working in lower level health facilities where there is no doctor, playing a critical role in providing PHC in the DRC [29]. The results show that supervisors in health facilities recognised that CBE-nurses have stronger competencies than OBE-nurses, especially in the areas of establishing professional communication, making decisions about health problems*,* and engaging in professional development. These results are aligned with the findings of the quantitative study in which the CBE- and OBE-nurses evaluated their own competencies using a DRC-specific self-assessment nurse competence scale [23]. The better performance by CBE-nurses in comparison with OBE-nurses has been proven in high- and upper-middle-income countries [30–32]. A review from China found that CBE-nurses performed better than OBE-nurses in terms of critical thinking, interpersonal communication, and professional development [33]. Our study showed that even in low-income countries such as the DRC, CBE can improve nursing care at health facilities.

Our study findings support the policy of the MoH in the DRC, which introduced CBE into the pre-service education system to achieve UHC through PHC. It also encourages other resource-limited countries to confidently promote CBE. However, the progress to shift from OBE to CBE has been slow. Fifteen years after the issuance of a ministerial decree, only 21% of the nursing education institutions have introduced CBE in the DRC. This study identified that the simultaneous implementation of the two approaches causes multiple challenges in health facilities, such as the feud between OBE- and CBE-nurses and the provision of unstandardised nursing care. The MoH continues preparing and conducting annual graduation examinations with different questions for CBE- and OBE-trained students, which places a heavy burden on the MoH in terms of finances, time, and human resources.

Some sub-Saharan African countries face the same challenges in implementing policies once they are enforced [34]. The successful transition from OBE to CBE in Rwanda highlights the importance of medium- and long-term reform plans [12]. In the DRC, the ministerial decree from the MoH to introduce CBE in 2005 was not legally binding, and the enforcement thereof was left to the provincial governments in a decentralised system, relying on each province’s leadership and capacity. A well-planned, legally binding CBE reform strategy will be helpful to speed up the transition to CBE. The MoH could consider abolishing the national standardised final examinations for OBE-trained students by a certain year. Strengthening the legally binding accreditation system for nursing education institutions could also be considered to close poor-quality ones, such as those having no capacity to introduce CBE curricula.

This study also revealed the importance of approaching the continuing professional development (CPD) system in CBE reform. Until all education institutions introduce CBE and all clinical nurses who graduated with OBE retire from their work, CBE- and OBE-nurses will continue to work together in health facilities across the country. This makes each health facility struggle to systematically provide standardised nursing care, as this study has identified. CBE-nurses expressed their challenges in demonstrating their competencies at their workplace where most supervisors and colleagues do not know about CBE. In fact, some supervisors who lack an understanding of CBE consider the performance of CBE-nurses, such as careful decision-making by consulting with other staff, as a shortfall and indicative of not being able to make decisions by themselves. This implies that supervisors lack a thorough understanding of the new education approach and cannot properly evaluate staff performance. To transfer the results of CBE to clinical practice, it is critical that all health facility staff, including supervisors and OBE-nurses, have a thorough knowledge of CBE through the CPD system so that CBE-nurses can effectively demonstrate their acquired competence in clinical settings [31].

The clinical skills of CBE-nurses were identified as relatively weak by supervisors. FGDs supported this observation and contradicts the curriculum of CBE in the fourth year which allocates longer hours in situational simulation and clinical training than the OBE curriculum (2498 h vs 2300 h) [23]. One of the reasons for this weakness could be the inadequate quality of clinical training provided for nurse students in health facilities, which is an issue in nursing education in many lower income countries [35–38]. In the DRC, provincial health departments have a role to monitor secondary nursing education institutions three times a year. However, no concrete monitoring tool for the provincial department exists to assess the quality of clinical training at health facilities. In the medium- and long-term plans for CBE reform, a concrete strategy to monitor and improve clinical training should be included. The collaboration between nursing education institutions, healthcare facilities, and officers at health departments at provincial and district levels is key to ensure highly competent future nurses.

Strong MoH leadership is critical in the comprehensive medium- and long-term strategic plan for a smoother transition from OBE to CBE both in the pre-service and in-service education system. In the DRC, three separate departments in the MoH manage human resources. The Department of Human Resources for Health is responsible for the recruitment and placement of personnel, the Department of Health Science Education is responsible for the pre-service education of secondary nurses, and the Department of Continuing Education is responsible for CPD. Although CBE reform is one of the priorities in the MoH [7], the three departments have no concrete plan to proceed with the reform in synergy. It is imperative to strengthen the cooperation between the three departments to accomplish the MoH’s priority to provide quality PHC at lower-level health facilities through competent nurses.

This study has several limitations. Although a 360-degree evaluation approach with each nurse is a more comprehensive methodology [39, 40], being a low-income country with a vast territory and limited resources, it has been difficult for the MoH to apply it. The results from our study, using interviews and FGDs to carefully sampled nurses, are still informative for policy implications in the DRC; thus, our methodology could be useful for other resource-limited countries. This study did not explore the broader challenges and constraints such as the working environment, and possible solutions in the wider health system. A more comprehensive health system analysis could be considered in future studies.

## Conclusion

The supervisors recognised that CBE-nurses have stronger competencies in professional communication, making decisions about health problems, and engaging in professional development, which supports the MoH policy to expand CBE in nationwide pre-service education. However, challenges exist for supervisors to assure standardised care at health facilities with two types of nurses, and for CBE-nurses to fully demonstrate their competencies at health facilities where they are a minority. The development and implementation of a well-planned, legally binding, longer-term CBE reform strategy, including an approach to the CPD system with strong cooperation among the three departments of the MoH, would be the key to accelerating the provision of PHC by competent nurses.

### Supplementary Information

Supplementary Material 1.

Supplementary Material 2.

Supplementary Material 3.

## Data Availability

The datasets used and/or analysed during this study are available from the corresponding author on reasonable request.

## Abbreviations

CBE: Competency-based education
CPD: Continuing professional development
DRC: Democratic Republic of the Congo
FGD: Focus group discussion
GH: General hospital
HC: Health centre
MoH: Ministry of Public Health
NCGM: National Center for Global Health and Medicine
OBE: Object-based education
PHC: Primary healthcare
UHC: Universal health coverage
