# Supplementary material for: Clinical competency of nurses trained in competency-based versus objective-based education in the Democratic Republic of the Congo: a qualitative study
Source: Hum Resour Health. 2024 Jun 4;22:38. doi: 10.1186/s12960-024-00921-0 (PMC11151529; doi:10.1186/s12960-024-00921-0)
Supplement: Supplementary file 1 — Supplementary Material 1. [file 12960_2024_921_MOESM1_ESM.docx]

**Fiche d’entretien avec les Responsables du service infirmier (ayant des infirmiers formés en APO et APC).**

**Identification du participant**

- Nom de l’institution sanitaire : …………………………………………..
- Secteur d’activité : -Public -Privé lucratif - Privé confessionnel - Autres (à préciser)
- Zone de santé  : .............................................................
- Province : .............................................................
- Fonction  :……………………………………………..
- Rapport hiérarchique avec l’infirmier : Chef direct -Autre niveau hiérarchique
- Sexe  : …………………………………..
- Date de l’entretien : / /
- Heure de début  :………………………………

**Introduction**

Cher collègue,

Je m’appelle …………, je suis de la Direction de l’Enseignement des Sciences de santé. Je vous présente également ………………………………mon/ma collègue de service ; il m’assiste dans ce travail.

Comme vous le savez probablement, la Direction de l’Enseignement des Sciences de santé a initié depuis 2005 une reforme de programme de formation basée sur le développement des compétences professionnelles. Certaines écoles de formation infirmière du niveau secondaire ont déjà mis sur le marché de travail des infirmiers A2. C’est le cas ici chez vous de l’infirmière/infirmier……..

Notre visite s’inscrit dans le cadre du suivi de cette réforme pédagogique. Elle consiste à évaluer le pas franchi quant à la qualité des soins offerts aux populations par les infirmiers A2 issus de la réforme et à s’enquérir des nouveaux défis lancés à la réforme et en termes de compétences à mobiliser pour atteindre le changement.

Notre entretien va durer environ une heure. Il sera enregistré afin de nous permettre la retranscription et de noter le maximum d’informations que vous nous aurez fournies. Nous vous rassurons que tout restera anonyme ; les informations reçues de vous ne seront utilisés que dans le cadre de ce travail d’évaluation.

Enfin, nous voudrions bien vous rassurer que tous vos avis sont nécessaires. Ainsi, nous vous prions de vous exprimer en toute liberté et vous encourageons à donner des commentaires francs qui peuvent améliorer notre travail.

Etes-vous d’accord de participer à cette étude ?

Notre entretien va s’articuler autour de quatre points : (i) les aptitudes professionnelles des infirmiers issus du programme par compétences ; (ii) la perception des clients des soins qu’offrent ces infirmiers ; (iii) la valeur ajoutée de la réforme pédagogique et enfin (iv) les améliorations éventuelles à y apporter.

**Questions**

***Question 1* :** Depuis comment de temps occupez-vous le poste de responsable dans cet établissement ?

***Question 2* :** Au regard des exigences du métier d’infirmier et des besoins de santé de la population de votre zone de santé, que dites-vous de la manière de travailler des infirmiers A2 de votre formation sanitaire ?

***Question 3*** : A votre avis, en quoi un infirmier issu du programme axé sur les compétences diffère-t-il de celui formé dans l’APO ? (Habileté, aptitudes, maitrise de soi, attitudes…)

***Question 4* :** Comment la population apprécie-t-elle la qualité des soins et services offerts par l’infirmier issu de la réforme pédagogique qui travaille dans votre hôpital/centre de santé par rapport à celui de l’APO ?

***Question 5* :** Partant de cette expérience de réforme pédagogique et du niveau de compétences ses infirmiers, comment peut-on améliorer ou renforcer davantage la formation de l’infirmier A2 ?

***Question 6*** : Comment procédez-vous au recrutement ou embauche des nouveaux infirmiers dans votre formation sanitaire ?

***Question 7*** : Organisez-vous de façon régulière de formation continue dans votre établissement sanitaire ?

***Question 8*** : Quel type de formation continue organisez-vous ?

***Question 9*** : Quels thèmes avez-vous déjà développé pour renforcer les capacités des infirmiers dans l’offre des soins et services ?

***Question 10***: Avez-vous un commentaire sur les prestations des infirmiers A2 issus du programme de formation par compétences ?

Merci pour votre collaboration !

Heure de la fin : ………………………………….
